# Carotid Intima-Media Thickness at Age 30, Birth Weight, Accelerated Growth during Infancy and Breastfeeding: A Birth Cohort Study in Southern Brazil

**DOI:** 10.1371/journal.pone.0115166

**Published:** 2015-01-22

**Authors:** Rogério da Silva Linhares, Denise Petrucci Gigante, Fernando Celso Lopes Fernandes de Barros, Bernardo Lessa Horta

**Affiliations:** Postgraduate Program in Epidemiology, Universidade Federal de Pelotas, Pelotas, Rio Grande do Sul, Brazil; Federal University of Pelotas, BRAZIL

## Abstract

**Objective:**

To examine the relationship between carotid intima-media thickness (IMT) at age 30 and birth characteristics, growth during infancy, and breastfeeding duration, among subjects who have been prospectively followed since birth.

**Methods and Results:**

In 1982, all births in the city of Pelotas, southern Brazil, were identified and those children (n = 5,914) whose families lived in the urban area of the city have been followed and evaluated at several time points. The cohort participants were evaluated in 2012–13, and IMT was measured at the posterior wall of the right and left common carotid arteries in longitudinal planes using ultrasound imaging. We obtained valid IMT measurements for 3,188 individuals. Weight-for-age z-score (WAZ) at age 2 years, weight-for-height z-score (WHZ) at age 4, height-for-age z-score (HAZ) at 4 years, WAZ at age 4 and relative conditional weight at 4 years were positively associated with IMT, even after controlling for confounding variables. The beta-coefficient associated with ≥1 s.d. WAZ at age 2 (compared to those with a <–1 s.d.) was 3.62 μm (95% CI 0.86 to 6.38). The beta-coefficient associated with ≥1 s.d. WHZ at 4 (in relation to <–1 s.d) was 3.83 μm (95% CI 0.24 to 7.42). For HAZ at 4, the beta-coefficient for ≥1 s.d. in relation to <–1 s.d. was 4.19 μm (95% CI 1.14 to 7.25). For WAZ at 4, the beta-coefficient associated with ≥1 s.d. in relation to <–1 s.d. was 4.28 μm (95% CI 1.59 to 6.97). The beta-coefficient associated with conditional weight gain at age 2–4 was 1.26 μm (95% CI 0.49 to 2.02).

**Conclusion:**

IMT at age 30 was positively associated with WAZ at age 2 years, WHZ at age 4, HAZ at age 4, WAZ at age 4 and conditional weight gain at age 4 years.

## Introduction

Cardiovascular disease (CVD) is the leading cause of death worldwide.[1] In 2008, there were 17.3 million deaths from CVD, of which 7.3 million from myocardial infarction and 6.2 million from stroke, and over 80% of deaths from CVD occurred in low- and middle-income countries.[1] Myocardial infarction and stroke are strongly related to atherosclerotic disease.

Atherosclerosis is characterized by the gradual thickening of the intimal and medial layers in large and medium-sized arteries. The measurement of carotid intima-media thickness (IMT), which is the main marker of atherosclerosis, has been used in epidemiological studies.[2–5] IMT of large peripheral arteries can be measured using high-resolution two-dimensional ultrasound imaging. This method is non-invasive, relatively simple and does not involve radiation exposure. In addition, the ultrasound measurement of IMT has a good correlation with histological measures of the aorta and carotid arteries.[3]

With respect to the programming effect of early exposures on carotid IMT, most of the studies have failed to report an effect of low birth weight, [6–9] whereas there is some controversy on the association between breastfeeding and IMT[10–12]. Concerning, weight gain, it has been suggested that IMT is positively associated with catch-up growth. Oren et al^9^ found that IMT in early adulthood was higher among low birthweight subjects who showed exaggerated weight gain in the first 2 years. Skilton et al [13] reported that weight gain, height-adjusted weight gain, and change in weight-for-height z score in the first 18 months was positively associated with IMT at 8 years. Evelein et al [14], reported that an excess of weight gain in relation to length in the first 3 months of life was positively associated with IMT at 5 years. Whereas, Leunissen et al observed that IMT was higher among subjects who were born SGA but had normal height at adulthood.[15]

This study was aimed at assessing the relationship of mean IMT, with birth weight, breastfeeding duration, anthropometric measurements and growth during childhood in a cohort of young adults that have been followed since birth.

## Methods

In 1982, the three maternity hospitals in Pelotas, a southern Brazilian city, were visited daily and the births were identified. Those liveborns (n = 5,914) whose family lived in the urban area of the city were examined and their mothers interviewed. The refusal rate was less than 1%. In the perinatal study, information on maternal and child health, family socioeconomic conditions, and pregnancy complications were collected. These subjects have been followed-up for several times and further information on the study methodology has been published elsewhere.[16–18] The subjects were followed up during childhood in 1984 and 1986. At these follow-ups, the mothers or guardians were interviewed and the children weighed and measured.

In 2012–13, we tried to contact the cohort members, which were invited to visit the study clinic. They were asked to sign an informed consent form and then were interviewed, examined, and a blood sample was drawn. IMT was measured at the posterior wall of the right and left common carotid arteries in longitudinal planes using ultrasound imaging.[5] A 10-mm-long section of the common carotid artery was imaged proximal to the carotid bulb. Image data was analyzed using the Carotid Analyzer for Research (Medical Imaging Applications, MIA-LLC). It automatically calculated the mean value of 90 measurements (frames) taken in the 10-mm-long section studied.

Birth weight measurements were taken with a pediatric scale that was calibrated on a weekly basis. Gestational age was calculated from the last menstrual period and birth weight according to gestational z-score was estimated based on the mean birth weight and standard deviations for gestational age and sex of the reference population developed by Williams.[19] Information on duration of breastfeeding (in months) was gathered in all follow-ups during childhood. In the follow-up visits, z-scores for weight-for-age (WAZ), height-for-age (HAZ) and weight-for-height (WHZ) were calculated using sex and age specifics values of the WHO growth charts.[20] Concerning anthropometric evaluation in the 1984 and 1986 visits, children were weighed with portable calibrated scales and length/height was measured using portable stadiometers.

Conditional weight/height is the residual of linear regression of all previous measurements of weight/height. This analysis takes into consideration the correlation between weight or length gain in subsequent age ranges, as well as regression to the mean. [21] The regression equation for height at age 2 years consisted of birth weight while the regression equation for weight at age 2 included birth weight and height at age 2. Conditional height at age 4 years was estimated from birth weight and weight and height measures at age 2. Conditional weight at age 4 years was estimated from birth weight, weight at 2 and 4 years, and height at 2 years. Positive results indicated that a child grew more rapidly than expected during that period compared with their prior growth and the population growth. Conditional variables were expressed in z-scores. Conditional growth represents the deviation from the expected weight or height, compared to other individuals from the population.

Potential confounding variables included family income at birth, maternal schooling, maternal skin color, maternal age, maternal smoking during pregnancy, skin color and sex. Analysis of variance was used to compare difference between means and multiple linear regression to adjust for confounding variables. The estimates for childhood anthropometry and growth were also adjusted for birth weight according to age z-score. The estimates for conditional height and weight were also adjusted for height at 30 years, because height is positively associated with blood pressure and IMT is associated with blood pressure.[22]

The institutional review board at the Universidade Federal de Pelotas Medical School approved the study protocol and all participants signed a written consent prior to all interviews and assessments.

## Results

We evaluated 3,701 subjects at the 30-year follow-up, which added to the 366 known to have died, represented a follow-up rate of 68.8%. Measurements of IMT were taken for 3,380 participants and they were valid for 3,188, and for 192 subjects the MIA-LLC software was not able to analyze the images.

Table 1 shows that the 30-year follow-up rate was lower among male and those subjects whose family income at birth was higher than 6 minimum wages. Birth weight, duration of breastfeeding and maternal skin color were not associated with follow-up rate.

**Table 1:** Distribution of characteristics, interviews and measurements of carotid intima-media thickness in the original cohort participants at the follow-up in 2012.

**Variable**	**Followed in 2012 (% original cohort)**	IMT measurements (% participants in 2012) *
Sex		
Male	2010 (66.2)	1585 (89.2)
Female	2045 (71.1)	1603 (83.8)
Maternal skin color		
White	3299 (68.0)	2623 (86.4)
Non-white	755 (71.2)	564 (86.4)
Family income at birth in minimum wages		
≤1.0	857 (66.5)	628 (86.7)
1.1–3.0	1984 (71.1)	1558 (86.0)
3.1–6.0	758 (69.5)	623 (86.5)
>6.0	436 (60,8)	366 (87.8)
Birth weight (grams)		
<2500	385 (72.1)	223 (84.2)
2500–2999	970 (69.6)	735 (84.3)
3000–3499	1477 (66.5)	1215 (87.6)
3500–3999	988 (69.7)	824 (88.0)
≥4000	232 (67.3)	190 (86.8)
Prematurity (<37 weeks of gestation)		
No	3005 (68.6)	2431 (86.6)
Yes	220 (74.8)	148 (88.6)
Gestational age-adjusted birth weight z-scores (Williams)		
<–1.28	482 (69.2)	364 (86.5)
–1.28–0.00	1433 (67.5)	1135 (85.7)
>0.00	1306 (70.6)	1079 (87.8)
Duration of breastfeeding		
<1 month	817 (69.8)	646 (84.9)
1–2.9 months	972 (69.2)	799 (87.3)
3–5.9 months	874 (72.1)	730 (88.5)
6–8.9 months	355 (71.4)	300 (88.0)
9–11.9 months	148 (70.8)	114 (80.3)
12 months or more	610 (72.8)	501 (85.4)
Total	4056 (68.6)	3188 (86.1)

The 1982 Pelotas Birth Cohort, Brazil.

* The total represented the subjects who were interviewed in 2012–13 (n = 3701)

IMT: intima-media thickness

Birth weight was positively associated with IMT, but this association wasn’t observed even after controlling for potential confounding variables. On the other hand, prematurity, birth weight according to gestational age z-score and duration of breastfeeding were not associated with IMT. There was a positive association between duration of breastfeeding and IMT up to 11.9 months; a lower IMT was observed among those breastfed for more than 12 months. But a non-significant association was observed, after controlling for confounding variables (Table 2).

**Table 2:** Crude and adjusted analysis of the association of birth weight and breastfeeding with carotid intima-media thickness in the cohort participants at the follow-up in 2012.

		**Mean**	**Regression coefficient**	
Variable		IMT	Crude	Adjusted
	N	µm	β (95% CI)	β (95% CI)
Birth weight (grams)				(n = 2563)
<2500	223	581.6	Ref (0)	Ref (0)
2500–2999	735	581.6	–0.00 (–2.78 to 2.78)	0.66 (–2.55 to 3.87)
3000–3499	1215	582.1	0.50 (–2.15 to 3.15)	0.24 (–3.28 to 3.76)
3500–3999	824	583.1	1.51 (–1.24 to 4.25)	0.52 (–3.79 to 4.83)
≥4000	190	585.5	3.94 (0.35 to 7.53)	1.53 (–4.43 to 7.50)
Prematurity (<37 weeks of gestation)				(n = 2563)
No	2431	582.4	Ref (0)	Ref (0)
Yes	148	580.9	–1.47 (–4.40 to 1.45)	–1.62 (–4.50 to 1.25)
Gestational age-adjusted birth weight z-scores (Williams)				(n = 2563)
<–1.28	364	581.9	Ref (0)	Ref (0)
–1.28 to 0.00	1135	581.6	–0.36 (–2.44 to 1.72)	0.16 (–1.91 to 2.23)
>0.00	1079	583.2	1.25 (–0.85 to 3.34)	2.04 (–0.07 to 4.15)
Duration of breastfeeding				(n = 2484)
<1 month	646	582.1	Ref (0)	Ref (0)
1–2.9 months	799	582.3	0.16 (–1.77 to 2.08)	–0.09 (–2.11 to 1.94)
3–5.9 months	730	582.3	0.17 (–1.79 to 2.13)	–0.18 (–2.23 to 1.88)
6–8.9 months	300	582.8	0.68 (–1.86 to 3.22)	1.65 (–1.01 to 4.31)
9–11.9 months	114	585.8	3.69 (0.00 to 7.39)	2.97 (–0.94 to 6.87)
12 months or more	501	581.5	–0.58 (–2.74 to 1.59)	–1.01 (–3.30 to 1.27)

The 1982 Pelotas Birth Cohort, Brazil.

Adjusted for confounding variables: family income at birth; maternal skin color; maternal age at child birth; maternal education; maternal smoking; sex; and skin color

Table 3 shows that WAZ at age 2, WHZ at age 4, HAZ at age 4 and WAZ at age 4 were positively associated with IMT, even after controlling for confounding variables. With respect to growth in childhood, relative conditional weight in the first 2 years of life was not associated with IMT while relative weight gain at 4 years was positively associated with IMT. Suggesting, that relative weight gain from 2 to 4 years was associated with higher IMT. On the other hand, there was no association between height gain and IMT.

**Table 3:** Crude and adjusted analyses of the association of anthropometric measures of children and accelerated growth during infancy with carotid intima-media thickness in the cohort participants at the follow-up in 2012.

**Variable**		**Crude analysis**	**Adjusted analysis 1**
	N	β (95% CI)	β (95% CI)
Weight-for-height z-score at age 2			(n = 2348)
<–1	151	Ref (0)	Ref (0)
–1 to 0.99	1809	0.62 (–2.56 to 3.80)	1.12 (2.36 to 4.60)
≥1	961	3.29 (0.01 to 6.57)	2.63 (–1.00 to 6.25)
Height-for-age z-score at age 2			(n = 2348)
<–1	1105	Ref (0)	Ref (0)
–1 to 0.99	1590	–0.19 (–1.62 to 1.24)	0.35 (–1.24 to 1.93)
≥1	227	1.46 (–1.21 to 4.12)	2.19 (–0.72 to 5.10)
Weight-for-age z-score at age 2			(n = 2348)
<–1	402	Ref (0)	Ref (0)
–1 to 0.99	1937	0.30 (–1.72 to 2.32)	0.89 (–1.37 to 3.15)
≥1	582	4.08 (1.70 to 6.46)	3.62 (0.86 to 6.38)
Weight-for-height z-score at age 4			(n = 2300)
<–1	138	Ref (0)	Ref (0)
–1 to 0.99	1875	0.72 (–2.51 to 3.94)	1.75 (–1.68 to 5.18)
≥1	847	4.54 (1.18 to 7.89)	3.83 (0.24 to 7.42)
Height-for-age z-score at age 4			(n = 2301)
<–1	1046	Ref (0)	Ref (0)
–1 to 0.99	1626	0.24 (–1.20 to 1.68)	0.38 (–1.19 to 1.96)
≥1	189	4.10 (1.24 to 6. 96)	4.19 (1.14 to 7.25)
Weight-for-age z-score at age 4			(n = 2302)
<–1	458	Ref (0)	Ref (0)
–1 to 0.99	1961	0.76 (–1.14 to 2.66)	0.86 (–1.22 to 2.94)
≥1	443	5.03 (2.61 to 7.46)	4.28 (1.59 to 6.97)
			(n = 2175)
Conditional height gain at age 0–2	2189	0.30 (–045 to 1.04)	*0.86 (–0.09 to 1.80)
			(n = 2175)
Conditional height gain at age 2–4	2189	0.63 (–0.11 to 1.37)	*0.43 (–0.39 to 1.25)
			(n = 2175)
Conditional weight gain at age 0–2	2189	0.69 (–0.06 to 1.44)	*0.67 (–0.06 to 1.41)
			(n = 2175)
Conditional weight gain at age 2–4	2189	1.51 (0.74 to 2.28)	*1.26 (0.49 to 2.02)

The 1982 Pelotas Birth Cohort, Brazil.

Adjusted for confounding variables 1: family income at birth; maternal skin color; maternal age at child birth; maternal education; maternal smoking; birth weight according to age z-score; sex and skin color

*Adjusted for 1 + height at 30 years

## Discussion

In this cohort that has been followed since birth, WAZ at age 2, WHZ at age 4, HAZ at age 4, WAZ at age 4 and relative weight gain at 4 years were positively associated with IMT, whereas we did not observe any association of weight gain in the first two years of life and linear growth (height or length gain) with IMT.

Information on birth weight was collected by the research team soon after delivery and examiners who had been carefully trained and standardized carried out the anthropometrical assessment in childhood. In the same token, data on breastfeeding duration was collected closed to the time of weaning. Ensuring, therefore, the quality of the data and minimizing the susceptibility to misclassification. Concerning selection bias, we followed up 68.8% of the cohort and managed to measure IMT among most of them (86.1%). Attrition rate was slightly higher among the wealthy subjects. On the other hand, in order to be the cause of the observed association, the losses should be related either to the nutrition in childhood and to IMT. Because this association is unlikely, we believe that the observed association was not due to selection bias.

A study conducted in Southern California with individuals aged 11 years also reported a positive association between birth weight and IMT, [7] whereas the Dutch ARYA study found that birth weight was negatively associated with IMT at 27 to 30 years of age. Our findings go in opposite direction to what has been proposed by Barker, i.e, low birth weight would be associated with a higher risk for cardiovascular diseases in adulthood. Therefore, we would expect a higher IMT among low birth weight subjects.[23] In the same token, we observed that WAZ and HAZ in childhood, were positively associated with IMT. Therefore, undernutrition in childhood was not associated with this variable, considered as a higher cardiovascular risk.

With respect to breastfeeding, a Dutch birth cohort (n = 306) observed among children aged 5 years that those who had been exclusively breastfed for 3 to 6 months had a carotid intima media thickness 21.1 µm (95% 5.0 to 37.2) higher than those who had never been breastfed, after controlling for confounding [10].. However, a population-based cohort study of Finnish young adults (n = 1,667) found no association of breastfeeding with IMT aged 24 to 39 years [11]. On the other hand, a British cohort study (aged 65 years) reported an inverse association between breastfeeding and IMT (difference —0.03 mm, 95% CI –0.07 to 0.01), but after controlling for confounding variables, a non-significant association was observed [12]. Our results stress the importance of controlling for confounding factors to avoid residual confounding. In our cohort, the duration of breastfeeding was inversely associated with socioeconomic condition and IMT. For this reason, the unadjusted analysis showed that breastfeeding was positively related to IMT and after controlling for several socioeconomic and demographic variables, the association disappeared.

Concerning weight gain, Skilton et al observed that weight gain from 0 to 18 months of age, was positively associated with carotid extra-medial thickness aged 8-years (11 mm per kg length-adjusted weight gain 95% CI 3 to 18), indicating that the alterations to the vasculature associated with excessive early postnatal growth likely include arterial adventitial thickening [24]. However, our study no found association between weight gain from 0 to 2 years and IMT at age 30. In another analyses, Skilton et al reported that weight gain, height-adjusted weight gain, and change in weight-for-height z score in the first 18 months was positively associated with IMT at 8 years [13]

Similarly to other studies, [25] we found that the adverse long-term effects of accelerated growth in infancy depend on the timing that growth acceleration occurs. IMT was not associated to early weight gain but to relative weight gain from 2 to 4 years of age. On the other hand, linear growth was not related to IMT. Taking together the evidence from our study with that from others ones reporting that early weight gain has a long-term benefit on human capital [26, 27] and cardiovascular risk factors, whereas late weight gain has no effect on human capital and increase cardiovascular risk factors.[25, 28] Therefore, early growth should be stimulated, but relative weight after the first two years should be prevented. On the other hand, promotion of linear growth does not seem to increase the risk of cardiovascular disease.

## References

[1] Mendis S, Puska P, Norrving B, World Health Organization, World Heart Federation, et al (2011) Global atlas on cardiovascular disease prevention and control. World Health Organization, Geneva 155 p.

[2] O’Leary DH, Polak JF, Kronmal RA, Manolio TA, Burke GL, et al (1999) Carotid-Artery Intima and Media Thickness as a Risk Factor for Myocardial Infarction and Stroke in Older Adults. New England Journal of Medicine 340(1):14–22. 10.1056/NEJM199901073400103 9878640

[3] Pignoli P, Tremoli E, Poli A, Oreste P, Paoletti R (1986) Intimal plus medial thickness of the arterial wall: a direct measurement with ultrasound imaging. Circulation 74(6):1399–406. 10.1161/01.CIR.74.6.1399 3536154

[4] Amato M, Montorsi P, Ravani A, Oldani E, Galli S, et al (2007) Carotid intima-media thickness by B-mode ultrasound as surrogate of coronary atherosclerosis: correlation with quantitative coronary angiography and coronary intravascular ultrasound findings. European Heart Journal 28(17):2094–101. 10.1093/eurheartj/ehm244 17597051

[5] Touboul PJ, Hennerici MG, Meairs S, Adams H, Amarenco P, et al (2012) Mannheim Carotid Intima-Media Thickness and Plaque Consensus (2004–2006–2011). Cerebrovascular Diseases 34(4):290–6. 10.1159/000343145 23128470 PMC3760791

[6] Hovi P, Turanlahti M, Strang-Karlsson S, Wehkalampi K, Järvenpää A-L, et al (2011) Intima-Media Thickness and Flow-Mediated Dilatation in the Helsinki Study of Very Low Birth Weight Adults. Pediatrics 127(2):e304–e11. 10.1542/peds.2010-2199 21262880

[7] Dratva J, Breton CV, Hodis HN, Mack WJ, Salam MT, et al (2012) Birth Weight and Carotid Artery Intima-Media Thickness. The Journal of Pediatrics 162(5):906–11. e1–2 10.1016/j.jpeds.2012.10.060 23260106 PMC4030536

[8] Gale CR, Ashurst HE, Hall NF, MacCallum PK, Martyn CN (2002) Size at birth and carotid atherosclerosis in later life. Atherosclerosis 163(1):141–7. 10.1016/S0021-9150(01)00760-2 12048132

[9] Oren A, Vos LE, Uiterwaal CSPM, Gorissen WHM, Grobbee DE, et al (2004) Birth weight and carotid intima-media thickness: new perspectives from the atherosclerosis risk in young adults (ARYA) study. Annals of epidemiology 14(1):8–16. 10.1016/S1047-2797(03)00068-1 14664774

[10] Evelein AM, Geerts CC, Visseren FL, Bots ML, van der Ent CK, et al (2011) The association between breastfeeding and the cardiovascular system in early childhood. The American Journal of Clinical Nutrition 93(4):712–8. 10.3945/ajcn.110.002980 21310835

[11] Jarvisalo MJ, Hutri-Kahonen N, Juonala M, Mikkila V, Rasanen L, et al (2008) Breast feeding in infancy and arterial endothelial function later in life. The Cardiovascular Risk in Young Finns Study. Eur J Clin Nutr 63(5):640–5. 10.1038/ejcn.2008.17 18285807

[12] Martin RM, Ebrahim S, Griffin M, Smith GD, Nicolaides AN, et al (2005) Breastfeeding and Atherosclerosis: Intima-Media Thickness and Plaques at 65-Year Follow-Up of the Boyd Orr Cohort. Arteriosclerosis, Thrombosis, and Vascular Biology 25(7):1482–8. 10.1161/01.ATV.0000170129.20609.49 15890972

[13] Skilton MR, Marks GB, Ayer JG, Garden FL, Garnett SP, et al (2013) Weight Gain in Infancy and Vascular Risk Factors in Later Childhood. Pediatrics 131(6):e1821–e8. 10.1542/peds.2012-2789 23713097

[14] Evelein AMV, Visseren FLJ, van der Ent CK, Grobbee DE, Uiterwaal CSPM (2013) Excess Early Postnatal Weight Gain Leads to Thicker and Stiffer Arteries in Young Children. Journal of Clinical Endocrinology & Metabolism 98(2):794–801. 10.1210/jc.2012-3208 23284005

[15] Leunissen RWJ, Kerkhof GF, Stijnen T, Hokken-Koelega ACS (2012) Effect of Birth Size and Catch-Up Growth on Adult Blood Pressure and Carotid Intima-Media Thickness. Hormone Research in Paediatrics 77(6):394–401. 10.1159/000338791 22760117

[16] Victora CG, Barros FC (2006) Cohort Profile: The 1982 Pelotas (Brazil) Birth Cohort Study. International Journal of Epidemiology 35(2):237–42. 10.1093/ije/dyi290 16373375

[17] Barros FC, Victora CG, Horta BL, Gigante DP (2008) Metodologia do estudo da coorte de nascimentos de 1982 a 2004–5, Pelotas, RS. Revista de Saúde Pública 42:7–15. 19142340 10.1590/s0034-89102008000900003 PMC2671798

[18] Victora CG, Barros FC, Lima RC, Behague DP, Gonçalves H, et al (2003) The Pelotas birth cohort study, Rio Grande do Sul, Brazil, 1982–2001. Cadernos de Saúde Pública. 19:1241–56. 10.1590/S0102-311X2003000500003 14666206 PMC2841342

[19] Williams RL, Creasy RK, Cunningham GC, Hawes WE, Norris FD, et al (1982) Fetal growth and perinatal viability in California. Obstetrics and gynecology 59(5):624–32. 7070736

[20] Borghi E, de Onis M, Garza C, Van den Broeck J, Frongillo EA, et al (2006) Construction of the World Health Organization child growth standards: selection of methods for attained growth curves. Statistics in Medicine 25(2):247–65. 10.1002/sim.2227 16143968

[21] Keijzer-Veen MG, Euser AM, van Montfoort N, Dekker FW, Vandenbroucke JP, et al (2005) A regression model with unexplained residuals was preferred in the analysis of the fetal origins of adult diseases hypothesis. Journal of Clinical Epidemiology 58(12):1320–4. 10.1016/j.jclinepi.2005.04.004 16291478

[22] Regnault N, Kleinman KP, Rifas-Shiman SL, Langenberg C, Lipshultz SE, et al (2014) Components of height and blood pressure in childhood. International Journal of Epidemiology 43(1):149–59. 10.1093/ije/dyt248 24413933 PMC3937979

[23] Barker DJ (1990) The fetal and infant origins of adult disease. BMJ. 301(6761):1111 10.1136/bmj.301.6761.1111 2252919 PMC1664286

[24] Skilton MR, Sullivan TR, Ayer JG, Garden FL, Harmer JA, et al (2014) Weight gain in infancy is associated with carotid extra-medial thickness in later childhood. Atherosclerosis 233(2):370–4. 10.1016/j.atherosclerosis.2014.01.020 24530765

[25] Adair LS, Fall CHD, Osmond C, Stein AD, Martorell R, et al (2013) Associations of linear growth and relative weight gain during early life with adult health and human capital in countries of low and middle income: findings from five birth cohort studies. The Lancet 382(9891):525–34. 10.1016/S0140-6736(13)60103-8 PMC3744751 23541370

[26] Horta BL, Gigante DP, Osmond C, Barros FC, Victora CG (2009) Intergenerational effect of weight gain in childhood on offspring birthweight. International Journal of Epidemiology 38(3):724–32. 10.1093/ije/dyp168 19376883 PMC2689398

[27] Martorell R, Horta BL, Adair LS, Stein AD, Richter L, et al (2010) Weight Gain in the First Two Years of Life Is an Important Predictor of Schooling Outcomes in Pooled Analyses from Five Birth Cohorts from Low- and Middle-Income Countries. The Journal of Nutrition 140(2):348–54. 10.3945/jn.109.112300 20007336 PMC2806888

[28] Horta BL, Victora CG, Lima RC, Post P (2009) Weight gain in childhood and blood lipids in adolescence. Acta Pædiatrica 98(6):1024–8. 10.1111/j.1651-2227.2009.01247.x 19484844 PMC2688671

